# Evaluation of a new culture medium for isolation of nontuberculous mycobacteria from environmental water samples

**DOI:** 10.1371/journal.pone.0247166

**Published:** 2021-03-03

**Authors:** Kimberly J. Alexander, Jennifer L. Furlong, Julianne L. Baron, John D. Rihs, Dominic Stephenson, John D. Perry, Janet E. Stout

**Affiliations:** 1 Special Pathogens Laboratory, Pittsburgh, PA, United States of America; 2 Microbiology Department, Freeman Hospital, Newcastle upon Tyne, United Kingdom; 3 Department of Applied Sciences, Northumbria University, Newcastle upon Tyne, United Kingdom; 4 Department of Civil and Environmental Engineering, Swanson School of Engineering, University of Pittsburgh, Pittsburgh, PA, United States of America; Maria Curie-Sklodowska University, POLAND

## Abstract

Nontuberculous mycobacteria (NTM) are waterborne pathogens commonly found in building water systems where they are a primary concern to vulnerable patient populations and can cause severe disease. The recovery of NTM from environmental samples can be a laborious undertaking and current pre-treatment methods and selective media lack sensitivity. We explored the use of the highly selective Rapidly Growing Mycobacteria (RGM) medium for culturing NTM from environmental water samples compared to existing methods. In total, 223 environmental water samples, including potable and non-potable water, were cultured for NTM using three culture media. In addition to direct culture on RGM medium, each sample was cultured on Middlebrook 7H10 medium and Mitchison 7H11 medium after pre-treatment with 0.2M KCl-HCl. Additionally, 33 distinct species of NTM were inoculated onto RGM medium and 7H10 medium in parallel to directly compare their growth. The use of RGM medium alone without pre-treatment provided a sensitivity (91%) comparable to that offered by culture on both 7H10 and 7H11 with acid pretreatment (combined sensitivity; 86%) with significantly less overgrowth and interference from other organisms on RGM medium. The average concentration of NTM observed on RGM medium alone was comparable to or greater than the NTM concentration on either medium alone or combined. Thirty-three species were examined in parallel and all tested strains of 27 of these species successfully grew on RGM medium, including 19 of 21 from the CDC’s healthcare-associated infections species list. RGM medium was successful at recovering environmental NTM without a pre-treatment, greatly reducing labor and materials required to process samples. Simplification of culture processing for environmental NTM will allow for a better assessment of their presence in building water systems and the potential for reduced exposure of susceptible populations.

## Introduction

Nontuberculous mycobacteria (NTM) are opportunistic waterborne pathogens that can be frequently found in drinking water systems where they may pose a threat to vulnerable patient populations, especially in healthcare environments [1]. In the United States the annual incidence of NTM disease is thought to be 3.2–9.8 per 100,000 population [2] and the associated economic burden of hospitalization has been estimated to be $435 million per year [3]. There are over 150 known species of NTM and infection from pathogenic strains is increasing in frequency with the potential to cause severe infection in those most vulnerable [4, 5]. *Mycobacterium* species have characteristics that make them difficult to control in building water systems even with on-site secondary disinfection. One of the species most relevant to human infection, *M*. *avium*, can grow at a wide range of temperatures, oxygen levels, pH, and salinity conditions and is highly resistant to both ozone and chlorine-based disinfectants [1, 6].

NTM can be recovered from a variety of engineered environments including aerosols from pools and spas, ice machines, potable water, instruments in healthcare settings, and in biofilms in water distribution systems [5, 6]. Human disease most commonly occurs from exposure to potable water sources and, in healthcare environments, can be caused by at least 17 NTM species or complexes including *M*. *abscessus* clade, *M*. *avium* complex, *M*. *chelonae*, and *M*. *mucogenicum* clade [6, 7]. NTM infections can be classified into four clinical presentations including chronic lung disease, lymphadenitis, cutaneous disease, and disseminated disease [5].

Currently, isolation and recovery of NTM from water using culture-based methods is an arduous process. Due to the diverse bacterial communities that exist in environmental samples, methods used for clinical samples are not commonly used for environmental samples [8]. Decontamination agents such as sodium hydroxide (NaOH), oxalic acid, and cetylpyridinium chloride (CPC) have all successfully been used to treat environmental samples for the recovery of NTM [9], however their use and concentration are variable among laboratory protocols and can result in differential isolation of species [10]. Elimination of non-mycobacterial species in a sample allows for the detection of NTM present in a previously overgrown sample. Although often successful at reducing non-mycobacterial growth, these decontamination methods have shown reduced recovery of some NTM species, as well as killing of others [9, 10].

Middlebrook 7H10 medium with OADC (oleic acid-albumin-dextrose-catalase) supplement is widely used for culture as it supports the growth of the vast majority of mycobacteria, however its lack of selectivity means that cultures may be frequently overgrown by other bacteria or fungi, if these remain viable after decontamination. To combat this problem, more selective variations of Middlebrook agar have been developed such as Mitchison 7H11 selective agar that contains carbenicillin, polymyxin B, amphotericin B, trimethoprim and malachite green as selective agents [11]. Culture on *Burkholderia cepacia* selective agar (BCSA) has been shown to reduce non-mycobacterial growth but has also been shown to be selective against some NTM species [12].

To avoid the toxic effects of chemicals that are used for sample decontamination, Preece et al. designed a highly selective agar medium, which they designated RGM medium as it was initially optimized for the isolation of rapidly growing mycobacteria (RGM) [13]. This medium allows the direct culture of clinical samples without the requirement for specimen decontamination. RGM medium has been used in several studies to isolate NTM from sputum samples of patients with cystic fibrosis (CF) and it afforded a higher sensitivity than traditional methods used for culture of acid-fast bacilli (AFB). In these previous studies, the standard AFB culture methods included both automated liquid culture and culture on Löwenstein Jensen medium [14, 15]. Notably, there is almost complete inhibition of yeasts, fungi and Gram-positive bacteria by RGM medium, as well as the vast majority of Gram-negative species [13].

Given the demonstrated utility of this medium for isolation of NTM from complex polymicrobial clinical samples, we decided to assess its ability to isolate NTM from potable and non-potable water samples. The first part of our evaluation involved culturing 223 water samples directly onto RGM medium (without sample decontamination) and examining the recovery of NTM in comparison with standard methods employed in our laboratory. Secondly, we inoculated various target species of NTM onto RGM medium and Middlebrook 7H10 medium used routinely in our laboratory to isolate NTM.

## Materials and methods

### Culture media and reagents

RGM medium was prepared by the Microbiology Department, Freeman Hospital, Newcastle upon Tyne, UK as previously described [16]. After quality assessment, it was then shipped to the Special Pathogens Laboratory (Pittsburgh, PA, United States) and used within a remaining shelf life of at least seven weeks. Middlebrook 7H10 agar and Mitchison 7H11 agar were obtained from Remel, Inc. (Lenexa, KS). KCl and HCl were obtained from Fisher Scientific (Waltham, MA).

### Sample processing

This study tested 223 environmental samples from varying locations in North America, collected and submitted to our laboratory from October 18, 2019 to June 1, 2020. Water samples were provided in high-density polyethylene bottles with enough sodium thiosulfate present to neutralize 20 ppm of chlorine. The samples were either 10X concentrated by filtration of 100 mL using a 0.2 μm Whatman Nucleopore track-etched polycarbonate membrane (Cytiva, Buckinghamshire, UK) placed into 10 mL of sterile DI water or, in the case of swab samples, they were expressed into 2 mL of sterile DI water. After concentration or expression, each sample was thoroughly vortexed and then processed using two different methods: 1) 100 μL of each sample was plated directly onto RGM medium and 2) each sample was pre-treated for 3 minutes with a 1:1 ratio of 0.2M KCl-HCl then 100 μL was plated on both Middlebrook 7H10 and Mitchison 7H11 media. Thus, the inoculum for RGM medium (100 μL) was directly equivalent to the cumulative inoculum used for the standard method (100 μL of a 1:1 dilution on each of two culture plates). Plates were sealed with parafilm to prevent moisture loss and incubated in a humidified incubator at 30°C ± 0.5°C for six weeks.

### Culture examination

Cultures were examined weekly during the six-week incubation. For each media type, all colonies considered possible NTM were sub-cultured onto Middlebrook 7H10 agar. After the sub-cultured colonies had sufficient growth, each colony type was confirmed as AFB by Kinyoun stain obtained from Remel, Inc. (Lenexa, KS) and identified by matrix assisted laser desorption ionization-time of flight mass spectrometry (MALDI-TOF MS) per manufacturer’s instructions (Bruker Scientific LLC, Billerica, MA). Counts of non-*Mycobacterium* colonies were also evaluated and up to three distinct colony morphologies from each sample were subjected to MALDI-TOF MS to obtain identification, where possible. Samples were classified as “overgrown” if they contained fungal growth that covered the entire plate or if there were enough non-AFB that the presence of NTM was unable to be determined.

### Known-strain comparison

Thirty-three species (45 isolates) of NTM were streaked side by side to directly compare growth on RGM medium compared to non-selective Middlebrook 7H10. These isolates were obtained from SPL’s stock culture collections (environmental sources), bioMérieux (clinical sources), and the American Type Culture Collection (ATCC), where noted. These isolates were identified using either 16S rRNA/*rpoB* gene sequencing or MALDI-TOF MS to confirm their speciation. These isolates included representatives of 21 species or complexes from the CDC’s list of NTM causing healthcare-associated infections [7] to evaluate the growth of clinically relevant species. Material from a single colony of NTM was isolation streaked, using the four-quadrant method, to each medium in parallel using a 1μL disposable loop. Plates were sealed with parafilm to prevent moisture loss and incubated in a humidified incubator at 30°C ± 0.5°C for up to six weeks. Plates were assessed weekly for presence/absence of growth.

### Statistical analyses

Pearson’s Chi-Square test and Fisher’s Exact Test were used to compare the proportion of positive samples by processing method and media type. A one-way analysis of variance (ANOVA) for correlated samples was performed to compare NTM concentrations by processing method and media type. Post-hoc Tukey’s honest significant difference (HSD) was performed to compare individual means. Plate counts of >300 colonies, yielding concentrations of >300 CFU/mL, were capped at 300 to calculate the means to reduce the effect of overly influential CFU counts on the statistics derived. A p-value of ≤ 0.05 was considered to be a statistically significant difference.

## Results

The sample set consisted of 86% potable samples and 14% non-potable samples. The 191 potable samples included 161 point-of-use outlets such as showers and faucets (84.3% of potable samples), 22 ice machines (11.5%), 3 hot water tanks (1.6%), and 5 swabs from potable sources (2.6%). The 32 non-potable sources included 19 cooling towers (59.4% of non-potable samples), 10 decorative fountains (31.2%), 1 hot tub/spa (3.1%), and 2 clinical heater-cooler devices (6.3%). The prevalence of NTM among these samples from a combination of all methods was 45%.

**Fig 1:**
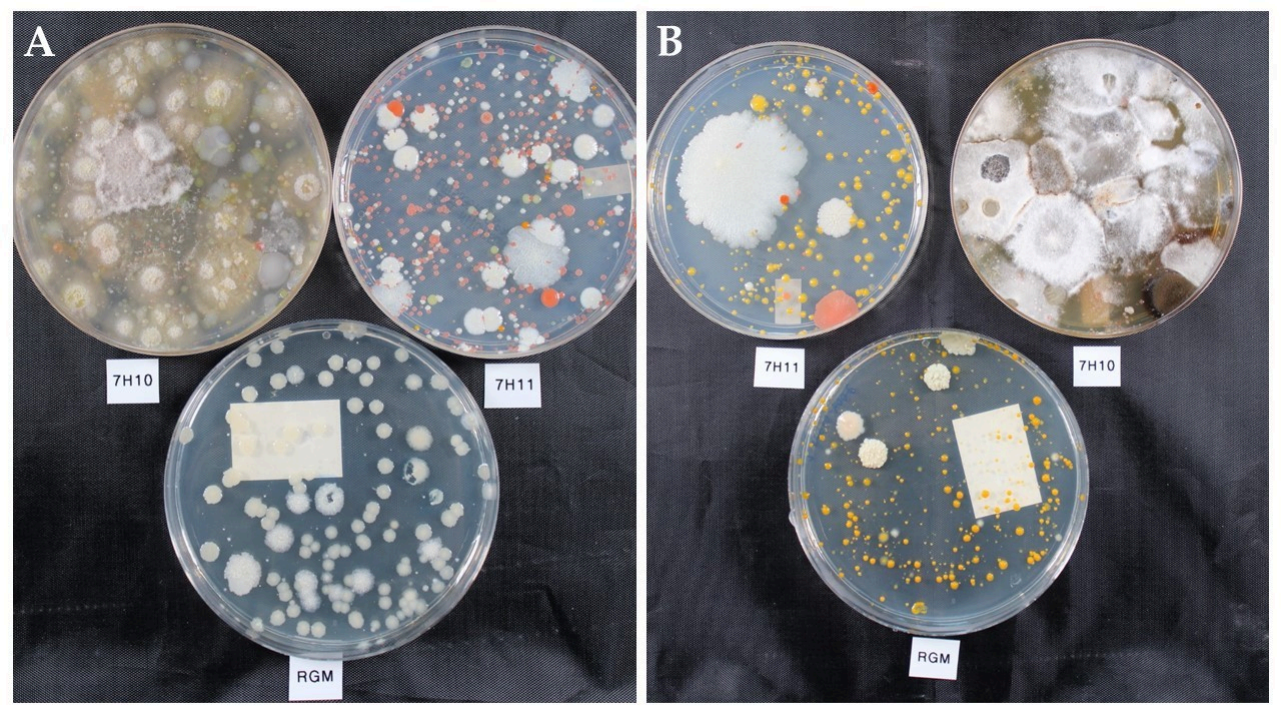
Visual comparison of two environmental water samples plated on 7H10 and 7H11 after acid treatment and RGM medium directly after six weeks of incubation. The 7H10 and 7H11 plates grew many and varied microbial species, however RGM medium selected for only NTM A) white, beige colonies and B) white, beige and yellow-orange colonies.

There was a significant difference among the proportion of total samples positive depending on the media and treatment when comparing individual media types (p = 0.05) (Table 1). When the combination of 7H10 + 7H11 was included in the comparison there was no significant difference in the proportions (p = 0.07). RGM medium had the highest percent positivity (41%) which by pairwise comparison was significantly higher than the 7H11 (30%) but not 7H10 (32%) or 7H10 + 7H11 (39%). There were also significant differences in the proportions of samples that were overgrown (p < 0.001) and those with non-mycobacteria present (p < 0.001) among the media types (Table 1). All three media types alone had statistically significantly different proportions of overgrowth from each other with 7H10 having the most at 17%, 7H11 in the middle with 9%, and RGM medium the least with 0%. The combination of 7H10 + 7H11 had the highest percentage of samples where at least one medium was overgrown (18%). In 8% of samples the combination of both 7H10 and 7H11 media was overgrown, which was also significantly higher than RGM medium. RGM medium had significantly fewer samples with non-NTM present (0.4%) than either 7H10 (46%) or 7H11 (37%) alone (p < 0.001) (Table 1). The combination of 7H10 + 7H11 had the highest proportion of samples with non-NTM present (48%) which was significantly higher than the percentage on RGM medium (0.4%). This was visually evident when comparing the different media types as well. RGM medium typically only grew NTM species, whereas both 7H10 and 7H11 were observed to have non-NTM species and NTM colonies (7H11) or were overgrown (7H10) (Fig 1).

**Table 1:** Summary of positivity, overgrowth, and non-NTM Isolated on 7H10, 7H11, and RGM medium.

	0.2M KCl-HCl Treated			RGM Medium (# of samples)
	7H10 (# of samples)	7H11 (# of samples)	7H10 + 7H11^3^ (# of samples)	
**Total Sample NTM Positivity**^**1**^	32% (72)	30% (68)	39% (86)	41% (91)
**Overgrown Samples**^**1**^	17% (38)	9% (19)	8% (17)	0% (0)
**Samples with Non-mycobacteria Present**^**1**^	46% (103)	37% (83)	48% (106)	0.4% (1)
**Detection of NTM Positives**^**2**^	72% (72)	68% (68)	86% (86)	91% (91)
**Average concentration of NTM Positives**^**4**^	79.0 CFU/mL (± 12.0 CFU/mL)	65.5 CFU/mL (± 9.7 CFU/mL)	72.3 CFU/mL (± 9.2 CFU/mL)	102.6 CFU/mL (± 11.9 CFU/mL)

^1^Percentages out of 223 samples

^2^Percentages out of 100 total positives by any method

^3^Combined results of each sample processed on both 7H10 and 7H11 resulting in the same initial sample inoculum volume as RGM medium.

^4^Average concentration of NTM in 100 positive samples plus or minus the standard error of the mean.

When looking only at the 100 NTM positive samples by any method, the number of NTM positives detected was significantly different among the media types: 7H10 (72%), 7H11 (68%), and RGM medium (91%) (p = 0.05) (Table 1). However, when adding the comparison to 7H10 + 7H11 there was no difference among the four proportions (p = 0.07). Utilizing different combinations of media resulted in increased sensitivity however each of the combinations did not differ from each other (p = 0.224) and none was significantly different from RGM medium alone (p = 0.10). RGM medium with 7H10 identified 99%, RGM medium with 7H11 identified 94%, and 7H10 with 7H11 identified 86% of positive samples. The average concentration of NTM identified from the media types was statistically significantly different (p < 0.001) (Table 1). RGM medium alone had the highest concentration (102.6 CFU/mL) of mycobacteria, which was significantly higher than 7H11 alone (65.5 CFU/mL) (p < 0.01) and the combination of 7H10 and 7H11 (72.3 CFU/mL) (p < 0.01) but not 7H10 alone (79.0 CFU/mL). There were 14 instances where RGM medium had the only quantifiable number of NTM colonies of the 3 media types for that sample, with an average concentration of 109.7 CFU/mL on RGM medium (range 1 to >300 CFU/mL). In 9 samples, RGM medium was negative but either 7H10, 7H11, or both had NTM colonies present with an average concentration of 4.7 CFU/mL on the 7H10 and 7H11 combination (range 1 to 14).

From the 100 environmental samples positive for NTM by any testing method, 29 distinct species or groups of NTM were recovered (Table 2). *M*. *gordonae* was the most common species isolated from environmental samples using all testing methods and was recovered most often on RGM medium (Table 2). More mycobacterial species were identified on RGM medium (n = 154) than 7H10 (n = 92) or 7H11 (n = 91) with several species often being identified in the same water sample. *M*. *avium* was only isolated from RGM medium. The average concentration of *M*. *avium* in these 7 samples was 15.4 CFU/mL (range 1–78 CFU/mL). A subset of plates (n = 34) was examined for time to positivity on the plates and the final colony size. Time to positivity on RGM medium compared to 7H10 and 7H11 was the same for most *Mycobacterium* species isolated. While initial colony sizes observed on RGM medium were slightly smaller, final colony sizes were comparable for most *Mycobacterium* species isolated.

**Table 2:** Identification of mycobacterial species on 7H10, 7H11, and RGM medium.

Organism Identification	0.2M KCl-HCl Treated 7H10		0.2M KCl-HCl Treated 7H11		RGM Medium	
	Potable	Non-potable	Potable	Non-potable	Potable	Non-potable
*M*. *abscessus*	2	0	1	0	2	0
*M*. *abscessus/ chelonae*	1	0	1	0	1	0
*M*. *arcueilense/ peregrinum*	0	0	0	0	0	1
*M*. *arupense*	4	0	1	0	4	0
*M*. *avium*	0	0	0	0	7	0
*M*. *chelonae*	1	0	1	0	1	0
*M*. *chelonae/ salmoniphilum/ stephanolepidis*	5	7	8	13	6	15
*M*. *chimaera/ intracellulare* group	4	0	5	1	12	2
*M*. *fortuitum*	2	0	2	2	2	4
*M*. *fortuitum* complex	1	0	1	0	1	0
*M*. *franklinii*	3	0	3	0	4	0
*M*. *gordonae*	30	4	23	5	35	9
*M*. *gordonae/ paragordonae*	1	0	0	0	1	0
*M*. *hodleri*	0	0	0	2	0	0
*M*. *immunogenum*	1	0	1	0	1	0
*M*. *lentiflavum*	0	0	0	0	0	1
*M*. *llatzerense*	2	0	0	0	2	0
*M*. *mantenii*	1	0	0	0	2	0
*M*. *mucogenicum*	0	0	0	0	2	0
*M*. *mucogenicum/ phocaicum*	16	2	8	3	13	5
*M*. *paraffinicum*	1	0	1	0	5	0
*M*. *paragordonae*	2	0	2	0	5	0
*M*. *peregrinum*	0	0	0	0	1	1
*M*. *porcinum*	1	0	1	0	1	1
*M*. *salmoniphilum*	0	0	1	0	1	0
*M*. *salmoniphilum/stephanolepidis*	1	0	1	0	1	0
*M*. *senegalense/ fortuitum* complex	0	0	0	0	0	1
*M*. *stephanolepidis*	0	0	1	0	1	0
*Mycobacterium* species	0	0	2	1	0	3
**Total**	**79**	**13**	**64**	**27**	**111**	**43**

**Fig 2:**
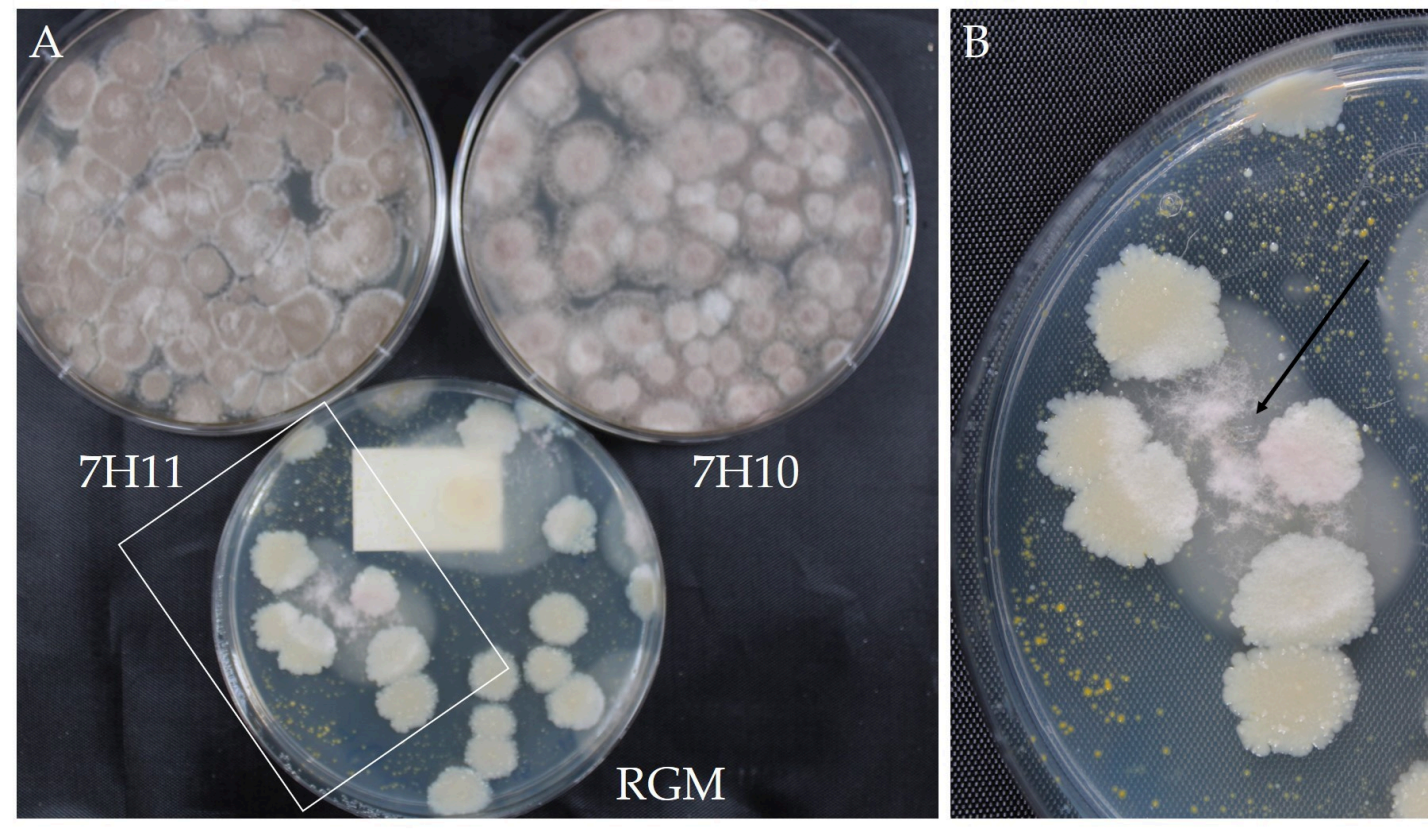
Visual comparison of *Purpureocillium lilacinum* on 7H10, 7H11, and RGM medium after six weeks of incubation. A) The 7H10 and 7H11 plates were overgrown with this fungus however the RGM medium partially inhibited the growth allowing recovery of NTM. B) Close up of inhibited growth of fungus from white box, black arrow shows the *P*. *lilacinum* colony. This was the only isolate of non-mycobacteria recovered from 223 environmental samples using RGM medium.

Colonies of non-mycobacteria were most frequently identified and grew in the highest number on 7H10. Gram-negative rods were identified more frequently from acid treated samples on both 7H10 and 7H11 (Table 3). Multiple *Methylobacterium* species were isolated from these water samples. During this study only one non-mycobacteria species was isolated on RGM medium and was identified as *Purpureocillium lilacinum*. This fungus overgrew the 7H10 and 7H11 media but still allowed for a readable plate on RGM (Fig 2).

**Table 3:** Identification of non-mycobacterial species on 7H10, 7H11, and RGM medium.

Organism Identification	0.2M KCl-HCl Treated 7H10		0.2M KCl-HCl Treated 7H11		RGM Medium	
	Potable	Non-potable	Potable	Non-Potable	Potable	Non-potable
*Afipia broomeae*	3	0	3	0	0	0
*Afipia massiliensis*	1	0	1	0	0	0
*Citrobacter* species	1	0	1	0	0	0
*Cupriavidus metallidurans*	1	0	0	0	0	0
Fungal overgrowth	3	11	1	2	0	0
*Gluconoacetobacter liquefaciens*	1	0	1	0	0	0
*Gordonia sputi*	1	0	0	0	0	0
Unidentified Gram-negative rods	44	5	35	2	0	0
*Methylobacterium extorquens*	1	0	1	0	0	0
*Methylobacterium fujisawaense*	10	0	8	0	0	0
*Methylobacterium hispanicum*	1	0	1	0	0	0
*Methylobacterium organophilum*	8	1	9	2	0	0
*Methylobacterium radiotolerans*	1	1	1	1	0	0
*Methylobacterium rhodesianum*	6	0	6	0	0	0
*Methylobacterium rhodinum*	1	0	1	0	0	0
*Methylobacterium* species	8	11	11	14	0	0
*Microbacterium maritypicum*	0	0	0	1	0	0
*Microbacterium oxydans*	1	0	0	0	0	0
*Nocardia amikacinitolerans*	0	1	0	0	0	0
*Nocardia cyriacigeorgica*	0	1	0	0	0	0
*Ochrobactrum anthropi*	0	1	1	0	0	0
*Purpureocillium lilacinum* (Fungus)	1	0	1	0	1	0
*Ralstonia pickettii*	1	0	1	0	0	0
*Reyranella massiliensis*	1	0	1	1	0	0
*Rhizobium radiobacter*	1	0	0	0	0	0
*Sphingomonas aquatilis*	1	0	0	0	0	0
*Sphingomonas paucimobilis*	10	0	9	0	0	0
*Sphingomonas species*	1	0	0	0	0	0
*Sphingomonas wittichii*	1	0	0	0	0	0
*Stenotrophomonas maltophilia*	1	0	0	0	0	0
*Tsukamurella paurometabola*	0	2	0	1	0	0
**Total**	**110**	**34**	**93**	**24**	**1**	**0**

Of the 33 species cultured on Middlebrook 7H10 and RGM medium for direct comparison, 33 species grew on 7H10, while 28 species grew on RGM (Table 4). *M*. *chlorophenolicum*, *M*. *gadium*, *M*. *haemophilum*, *M*. *neoaurum*, *M*. *phlei*, and one strain of *M*. *xenopi* did not grow on RGM medium. None of these species was recovered from environmental water samples on any type of medium during this study. Twenty of the 21 strains of NTM on the CDC Healthcare-associated Infection (HAI) list successfully grew on RGM medium (Table 4). Time to positivity on RGM medium and 7H10 for known strains was comparable for most *Mycobacterium* species isolated. Initial colony size observed on RGM medium was slightly smaller, however final growth was equivalent at the end of 6 weeks.

**Table 4:** Comparison of known NTM strains on 7H10 and RGM medium.

Organism Name	Source	CDC HAI List?	7H10 Growth	RGM Medium Growth
*M*. *abscessus*	Environmental	Yes	+	+
*M*. *arupense*	Environmental	Yes	+	+
*M*. *aurum*	ATCC 23366	Yes	+	+
*M*. *aurum*	ATCC 23070	Yes	+	+
*M*. *aurum*	ATCC 21498	Yes	+	+
*M*. *avium*	ATCC 25291	Yes	+	+
*M*. *chelonae*	Environmental	Yes	+	+
*M*. *chimaera*	Environmental	Yes	+	+
*M*. *fortuitum*	Environmental	Yes	+	+
*M*. *gordonae*	Environmental	Yes	+	+
*M*. *haemophilum*	Clinical	Yes	+	-
*M*. *haemophilum*	Clinical	Yes	+	-
*M*. *immunogenum*	Environmental	Yes	+	+
*M*. *intracellulare*	ATCC 13950	Yes	+	+
*M*. *kansasii*	ATCC 25101	Yes	+	+
*M*. *mucogenicum*	Environmental	Yes	+	+
*M*. *nonchromogenicum*	Clinical	Yes	+	+
*M*. *nonchromogenicum*	Clinical	Yes	+	+
*M*. *parascrofulaceum*	Clinical	Yes	+	+
*M*. *parascrofulaceum*	Clinical	Yes	+	+
*M*. *phocaicum*	Environmental	Yes	+	+
*M*. *porcinum*	ATCC 33776	Yes	+	+
*M*. *scrofulaceum*	Clinical	Yes	+	+
*M*. *scrofulaceum*	Clinical	Yes	+	+
*M*. *simiae*	Clinical	Yes	+	+
*M*. *simiae*	Clinical	Yes	+	+
*M*. *simiae*	Clinical	Yes	+	+
*M*. *simiae*	Clinical	Yes	+	+
*M*. *smegmatis*	Clinical	Yes	+	+
*M*. *smegmatis*	Clinical	Yes	+	+
*M*. *xenopi*	Clinical	Yes	+	-
*M*. *xenopi*	Clinical	Yes	+	+
*M*. *chlorophenolicum*	Environmental	No	+	-
*M*. *franklinii*	Environmental	No	+	+
*M*. *gadium*	Environmental	No	+	-
*M*. *hodleri*	Environmental	No	+	+
*M*. *llatzerense*	Environmental	No	+	+
*M*. *mantenii*	Environmental	No	+	+
*M*. *neoaurum*	Environmental	No	+	-
*M*. *neoaurum*	Environmental	No	+	-
*M*. *palustre*	Environmental	No	+	+
*M*. *paraffinicum*	Environmental	No	+	+
*M*. *peregrinum*	Environmental	No	+	+
*M*. *phlei*	Environmental	No	+	-
*M*. *salmoniphilum*	Environmental	No	+	+

## Discussion

Opportunistic pathogens, such as NTM species, have adapted to grow and reside in potable premise plumbing systems and can even be selected for by common municipal disinfection methods including monochloramine treatment [17, 18]. Although there are many different methods for isolating NTM from both clinical and environmental samples, none of these methods offers ideal selectivity even with the laborious undertaking of decontamination steps. The presence of contamination by non-mycobacteria and the length of time required for NTM growth means there is a higher risk of NTM going undetected using typical environmental NTM testing methods. Current decontamination processes and selective media, such as 7H11, have also been shown to suppress the growth of some species of NTM [10, 19]. Both clinical and environmental samples processed for NTM often require methods or media with high selectivity to restrict the growth of contaminants that are often present in the sample [16].

In this study, we report the first evaluation of RGM medium for the recovery of NTM in environmental water samples. Out of the 223 samples tested, 191 samples were from a variety of potable sources and 32 samples were from non-potable environmental sources. A mixture of sample types was chosen to create a challenge set representative of environmental waters samples. Including a variety of potable and non-potable samples increased the likelihood of exposure to differing NTM species, as well as a wider variety of contaminating species. RGM medium detected more NTM positive samples than were found on either 7H10 or 7H11 alone after acid treatment and had the highest average concentration of NTM. However, in combination 7H10 and 7H11 acid treated cultures identified a comparable number of samples positive for NTM as RGM medium with no pre-treatment. Sensitivity was increased when the results from plating on RGM medium were combined with acid treated samples cultured on 7H10. This combination identified 99% of NTM positive environmental water samples detected by a combination of all methods used here, however this was not significantly improved compared to RGM medium alone (91%; p = 0.10). RGM medium recovered the highest concentration of NTM on average per sample which was significantly higher than 7H11 alone or the combination of 7H10 and 7H11, but not 7H10 alone. When RGM medium had no observed NTM, but at least one of the other media types did, the count was low on average (4.7 CFU/mL; range 1 to 14 CFU/mL), suggesting that this could have happened by chance based on plating. However, when only RGM medium recovered NTM, the average concentration was much higher (109.7 CFU/mL; range 1 to >300 CFU/mL). The risk of non-mycobacterial contamination was significantly reduced on environmental samples plated on RGM medium. Samples with high levels of contamination and overgrowth such as those from non-potable sources like cooling towers and decorative fountains, did not yield overgrown cultures on RGM medium but often did on 7H10 or 7H11 even after acid treatment. In samples with high concentrations of NTM on RGM medium only, the other media types were observed to be heavily contaminated with non-NTM organisms such that no NTM could be observed on these plates. The lack of selectivity of 7H10 and 7H11 even with acid pre-treatment can prevent the recovery of NTM. RGM medium contains a combination of selective agents that includes traditionally used agents such as colistin and amphotericin but also contains the novel selective agents fosfomycin and 9-chloro-9-[4-(diethylamino) phenyl]-9,10-dihydro-10-phenylacridine hydrochloride (C-390) [16]. Colistin inhibits the vast majority of *Pseudomonas* species and C-390 is highly effective at inhibiting the vast majority of other non-glucose-fermenting Gram-negative bacteria. These attributes have proven to be highly useful in the testing of water samples for NTM, with the complete elimination of Gram-negative bacteria that were recovered frequently on conventional agars despite decontamination (Table 3).

NTM have been identified in the built environment from sources ranging from potable distal outlets to non-potable sources such as cooling towers and decorative fountains. Studies of distal outlets in the United States show that NTM species frequently isolated include *M*. *avium*, *M*. *abscessus*, *M*. *chelonae*, *M*. *gordonae*, *M*. *intracellulare*, *M*. *mucogenicum*, and *M*. *phocaicum* [18, 20, 21]. These organisms, and many others, were also recovered in our study. Interestingly, a total of 7 samples were shown to contain *M*. *avium* and in each case this species was only recovered on RGM medium. The average concentration of *M*. *avium* in these samples was low overall, however, we would have expected growth on 7H10 and 7H11 for some of the higher concentrations, if there was no interference with non-NTM on these plates. The presence of non-NTM species on culture media may obscure the presence of *M*. *avium*, which can be a cause of clinically significant human infections. Furthermore, it is notable that over twice as many isolates of *M*. *chimaera*/*intracellulare* group were recovered on RGM medium compared with culture on any other medium. Serious infections associated with *M*. *chimaera* have been associated with acquisition via bioaerosols emitted from contaminated heater-cooler units during cardiopulmonary bypass [22]. Overall, more different species of NTM were isolated on RGM. This is likely because the lack of contamination on the RGM medium allowed for organisms in low concentrations to be recovered and often several species could be identified from a single water sample. Alternatively, the acid treatment may kill off certain species of NTM, particularly at low inocula. At least 31 different isolates of non-NTM microorganisms were identified on the 7H10 and 7H11 acid treated cultures. Many of these were unidentifiable by MALDI-TOF MS and were categorized as gram-negative rods. The presence of many different bacterial species in environmental water samples is well known though many of these organisms remain uncategorized and unstudied. We observed several expected waterborne bacteria including *Methylobacterium* species, *Sphingomonas* species, and *Stenotrophomonas maltophilia* [23, 24]. We identified one fungal species (*Purpureocillium lilacinum*) that was able to grow on RGM medium, though it was substantially inhibited. Formerly a *Paecilomyces* species, this organism has been found in municipal and hospital water samples, can be a member of the environmental water biofilm community [25, 26], and can cause disease in immunocompromised individuals [27].

Although this study evaluated RGM medium using environmental water samples, many of the NTM species isolated have clinical significance. Several types of NTM have been associated with infections or colonization of the respiratory tract in cystic fibrosis patients. In a study of respiratory samples from CF patients by Plongla et. al., *M*. *gordonae*, *M*. *immunogenum*, *M*. *mucogenicum*, *M*. *chelonae*, *M*. *avium* complex, and *M*. *abscessus* complex were isolated on RGM medium [14]. These species were all recovered from environmental samples on RGM medium during this study. Additionally, a study of pulmonary and extrapulmonary samples from patients also identified *M*. *gordonae* and *M*. *avium* frequently, as well as *M*. *intracellulare*, *M*. *kansasii*, and *M*. *peregrinum* [28]. During our study nine NTM species from the CDC list of mycobacteria causing HAIs [7] were isolated on RGM medium from environmental samples. These species include *M*. *abscessus*, *M*. *arupense*, *M*. *avium*, *M*. *chelonae*, *M*. *chimaera/intracellulare* group, *M*. *gordonae*, *M*. *fortuitum*, *M*. *immunogenum*, and *M*. *mucogenicum*.

We evaluated clinically and environmentally relevant species in the known-strain comparison experiment to assess growth on RGM medium. There were several strains of NTM that were unable to grow on RGM medium including *M*. *chlorophenolicum*, *M*. *gadium*, *M*. *haemophilum*, *M*. *neoaurum*, *M*. *phlei*, and one strain of *M*. *xenopi*. Some of this may be due to specific growth requirements of these species or the particular strain, as one strain of *M*. *xenopi* grew while another did not. We have previously identified *M*. *neoaurum* [15] and *M*. *chlorophenolicum* in clinical samples on RGM medium. There is evidence to suggest that water reservoirs are a likely source of *M*. *haemophilum* infection but despite this, clinical isolates have not been linked to environmental isolates [29]. This is likely due to the fastidious nature of *M*. *haemophilum* which requires the addition of blood or ferric ammonium citrate to grow [30]. *M*. *xenopi* may also frequently be missed by culture as its optimal growth temperature (43°C) is often much higher than that used for culture of environmental samples [30].

None of these organisms were found in water samples during our study so we were unable to evaluate their growth from environmental sources. However, at least some of these species including *M*. *haemophilum* [1], *M*. *neoaurum* [31], *M*. *phlei* [32] and *M*. *xenopi* [33] have been isolated from water samples by others. Only *M*. *haemophilum* and *M*. *xenopi* were noted by the CDC as environmental organisms of concern for HAIs. Utilization of 7H10 after acid treatment may improve recovery of these other organisms however, we have shown that cultures on 7H10 are highly susceptible to overgrowth by non-NTM.

Human disease due to NTM is increasing [34] and being linked to organisms found in the environment, including *M*. *avium* and *M*. *intracellulare*. These organisms can be aerosolized in environmental sources such as showers, causing pulmonary disease [10, 35]. In fact, NTM species have been found to be preferentially aerosolized from showers [23], leading to the potential for an increased risk of exposure [36]. The use of RGM medium for mycobacterial cultures offers the selectivity necessary for isolating NTM from both clinical and environmental samples, while enabling one-step, direct processing. Its lack of contamination without the suppression of the majority of environmentally relevant NTM species can lead to more accurate culture results. Addition of acid treatment and plating the environmental water sample on 7H10 agar, could be used to recover more NTM positive samples and to ensure no strain specific growth is missed. The noted increase in severe human disease caused by *Mycobacterium* species and the frequency of its recovery in premise plumbing makes it increasingly important to monitor environmental water samples for NTM. This will allow for mitigation of the potential risk of exposure to susceptible individuals, especially in the healthcare environment. The ease of use of RGM medium and its accuracy in recovering and detecting environmental mycobacteria should improve the effectiveness of this monitoring. Further studies are needed to continue to evaluate the usefulness of RGM medium for environmental samples in different geographical locations, however, the results of this study and previous clinical evaluations suggest that RGM medium can add significant value to current laboratory NTM processing methods.

## Supporting information

S1 Data (XLSX)
